# Characterization of spatiotemporal electroactive anodic biofilm activity distribution using 1D simulations

**DOI:** 10.1038/s41598-022-09596-w

**Published:** 2022-04-07

**Authors:** Pierre Belleville, Gerard Merlin, Julien Ramousse, Jonathan Deseure

**Affiliations:** 1 Univ. Grenoble Alpes, Univ. Savoie Mont Blanc, CNRS, Grenoble INP Institute of Engineering, LEPMI, 38000 Grenoble, France; 2 Univ. Savoie Mont-Blanc, CNRS, LOCIE, UMR 5271, Polytech Annecy, Chambéry, bât. Helios, 60 rue du lac Léman, Savoie Technolac, 73370 Le Bourget du Lac, France

**Keywords:** Microbiology, Environmental sciences, Engineering, Mathematics and computing

## Abstract

Activity distribution limitation in electroactive biofilm remains an unclear phenomenon. Some observations using confocal microscopy have shown notable difference between activity close to the anode and activity at the liquid interface. A numerical model is developed in this work to describe biofilm growth and local biomass segregation in electroactive biofilm. Under our model hypothesis, metabolic activity distribution in the biofilm results from the competition between two limiting factors: acetate diffusion and electronic conduction in the biofilm. Influence of inactive biomass fraction (i.e. non-growing biomass fraction) properties (such as conductivity and density) is simulated to show variation in local biomass distribution. Introducing a dependence of effective diffusion to local density leads to a drastic biomass fraction segregation. Increasing density of inactive fraction reduces significantly acetate diffusion in biofilm, enhances biomass activity on the outer layer (liquid/biofilm interface) and maintains inner core largely inactive. High inactive fraction conductivity enhances biomass activity in the outer layer and enhances current production. Hence, investment in extracellular polymer substance (EPS), anchoring redox components, is benefit for biofilm electroactivity. However, under our model hypothesis it means that conductivity should be two order lower than biofilm conductivity reported in order to observe inner core active biomass segregation.

## Introduction

Microorganism ability to convert chemical energy into electrical energy has been largely studied for these last decades thanks to a wide range of possible applications such as wastewater or pollutant treatment^1,2^, microsensor power supply, biosensors, nutrient recovery or microbial electrosynthesis^3^. Indeed, some bacteria can use solid electrode as an electron acceptor (bioanode) or an electron donor (biocathode) to provide catabolic energy to run their metabolism. Anode respiring bacteria (ARB) are able to develop a biofilm structure with a thickness varying from 30 to 150 µm mainly made of metabolically active bacteria surrounded by extracellular polymeric substance (EPS)^4^. EPS matrix plays multiple roles in biofilm structure, such as mechanical cohesion, stress resistance, biofilm auto-maintenance (by EPS hydrolysis)^5^, and redox activity which is essential for long range electron transfer^6^. Necessary conditions (electrode potential, pH, substrates composition and concentration) must be created to have a biofilm with a minimum of heterogeneity of its metabolic activity and therefore as electroactive as possible. It is thus necessary to understand and control the activity limitation phenomena due to charge transfers and the diffusion of the substrate, which are the main limiting factors identified^7^.

Extracellular electron transfer (EET) mechanisms in ARB communities remain a question of major interest in microbial electrochemistry field. The outer membrane cytochrome Z (OmcZ) has been identified as responsible for the final electron transfer from *Geobacter *sp. biofilm to the solid anode^8^. However, direct electron transfer (DET) to solid anode is not able to explain the high-density current observed in microbial fuel cell (MFC)^9,10^. The role of EPS, giving a long-range physical contact, between bacteria and electrodes and an anchoring place for cytochromes networks the best theory to explain high density current observed^11,12^. However, the mechanism taking place at molecular scale is controversial^13,14^. One model defends Red-Ox cytochromes network (superexchange) which can explain the limit biofilm growth (fully oxidized cytochromes on the outer layer)^15^ and the multiple c-type cytochromes production in ARB^16^. The other defends metal-like conductivity pili, which allows longer range transfer than the mean inter-cytochromes distance^17^. Models of couple parallel mechanisms have been proposed, considering a mechanistic stratification in the biofilm, cytochromes drive the inner core growth and pilus drive the outer layer growth^12^. The presence of transmembrane macromolecule, coupling piliA and OmcS, could confirm this approach^18^. Moreover, EPS composition has been identified as responsible of electron storage mechanisms and could explain anode dependance biofilm electroactivity^19,20^.

The use of computing simulations under various conditions seems the most relevant way to explore hypotheses on electroactivity distribution within the biofilm based on observations and experiments. So far, several electroactive biofilm growth models have been developed using external mediator diffusion transfer^21^**,** matrix conduction transfer^22^ or mixed electron transfer^23,24^. Matrix conduction transfer allows to link biomass growth to local donor concentration and local potential (using Nernst Monod law^22^). This model is easy to fit to experimental data^25^, using the midterm potential E_ka_ which expresses the biofilm ability to transfer electron under specific conditions (biofilm composition, anode potential). Therefore, this model has been largely reused and extended^23,26^. However, it cannot compute the conversion rate using a thermodynamic approach. By contrast, the approach of external mediator diffusion permits to separate local biomass growth (using a double Monod Law) from final electron transfer to anode (using a Butler-Volmer law)^27^. In addition, it allows calculation of catabolic energy available for biomass growth from thermodynamic approach^28^ and simulate cyclic voltammetry (CV) in turnover condition. However, this model requires several additional parameters such as redox mediator concentrations, reaction speed rate or local diffusion coefficient, which are difficult to determine from experiments.

In biofilm field, several models have been proposed to express extracellular matrix compounds and transformation rates^29,30^. Laspidou et al.^31^ made a critical review on EPS, inert and inactive biofilm production mechanism and proposed a unified theory incorporating growth-dependent EPS production, EPS hydrolysis into biodegradable product (maintenance) and inert residual material. In comparison, in anode respiring biofilm models, most authors separate acetate fed biofilm into two volume fractions: an active part and an inactive part. The active fraction relies on biomass growth from substrate oxidation whereas the inactive fraction pulls together others solid components (EPS, pilin, insoluble byproducts). Merkey et al*.*^32^ introduced an EPS production investment coefficient in ARB, reducing biomass growth and current production.

Phenomena of segregation of metabolic and electroactive activity indicating heterogeneity have been observed in the literature but activity distribution within the biofilm remains unclear. Studies by fluorescence live/dead test (LIVE/DEAD® BacLight™), that brings in light the membrane state and the metabolism activity, show either a high activity close to the electrode^33^ or on the outside part^34^ while running. This could be explained by various mechanisms, which limit electroactive bacteria growth and electron production^6^. Explanations have been proposed such as diffusion limitation in the inner core, long range electron transfer limitation or pH inhibition. We have to consider that this detection technique does not offer strong evidence concerning activity because BacLight™ test is based on propidium iodide which depends on the integrity of the cell membrane and membrane potential. It is very likely that this dye also flows into cell membrane during cell division yielding falsely declared dead cells^35^. The definition of the “viability state” of microorganisms has been a matter of confusion and discussion for decades and has not yet been solved. “Viability staining” or “vital staining techniques” have been and are still used to overcome the problem of distinguishing between live and dead microorganisms in biofilms^36^. It is important to note that cell viability and cell vitality represent two different aspects of cell functions, and both are required for the estimation of the real physiological state of a cell after exposure to various types of stressors and chemical or physical factors^37^. Moreover, the membrane potential is probably affected by reduction state of the cytochromes, substrate availability or redox gradients. Although that this detection technique may produce artefacts or false positives. Therefore, simulation screening cans highlight such phenomena under similar operating conditions.

The aim of this work is thus to propose a numerical model to emphasize the influence of inactive biomass (including EPS) properties on biofilm activity distribution. In this work, we have defined biofilm activity on a term of place where cell growth takes place. The biomass having this growth activity will be called the active biomass, the inactive biomass will therefore be localized in the places without growth. Current models do not account for the biofilm distribution phenomena at the anode that has appeared to remarkably affect the overall performance of MFC, which is considered one of the influencing factors of biofilm’s dynamic performance^38^.

Simulations using 1D modeling have been conducted in order to study the segregation of biofilm activity during its development such as influence of inactive biomass density; influence of the conductivity of inactive biomass and influence of pH change. In the first instance, a simple "ion/electron conduction" model which corresponds to a growth limited by the maximum fixed thickness was built. The model made it possible to analyze the sensitivity of key parameters and to calibrate the values from the literature data. To consider the density and conductivity properties of the biofilm EPS matrix, the initial model was improved to assess the influence of the density and conductivity of inactive biomass. Then, main electroactive biofilm growth limitation factors were simulated, i.e., limitation by substrate diffusion and limitation by charge transport.

## Model description

**Figure 1:**
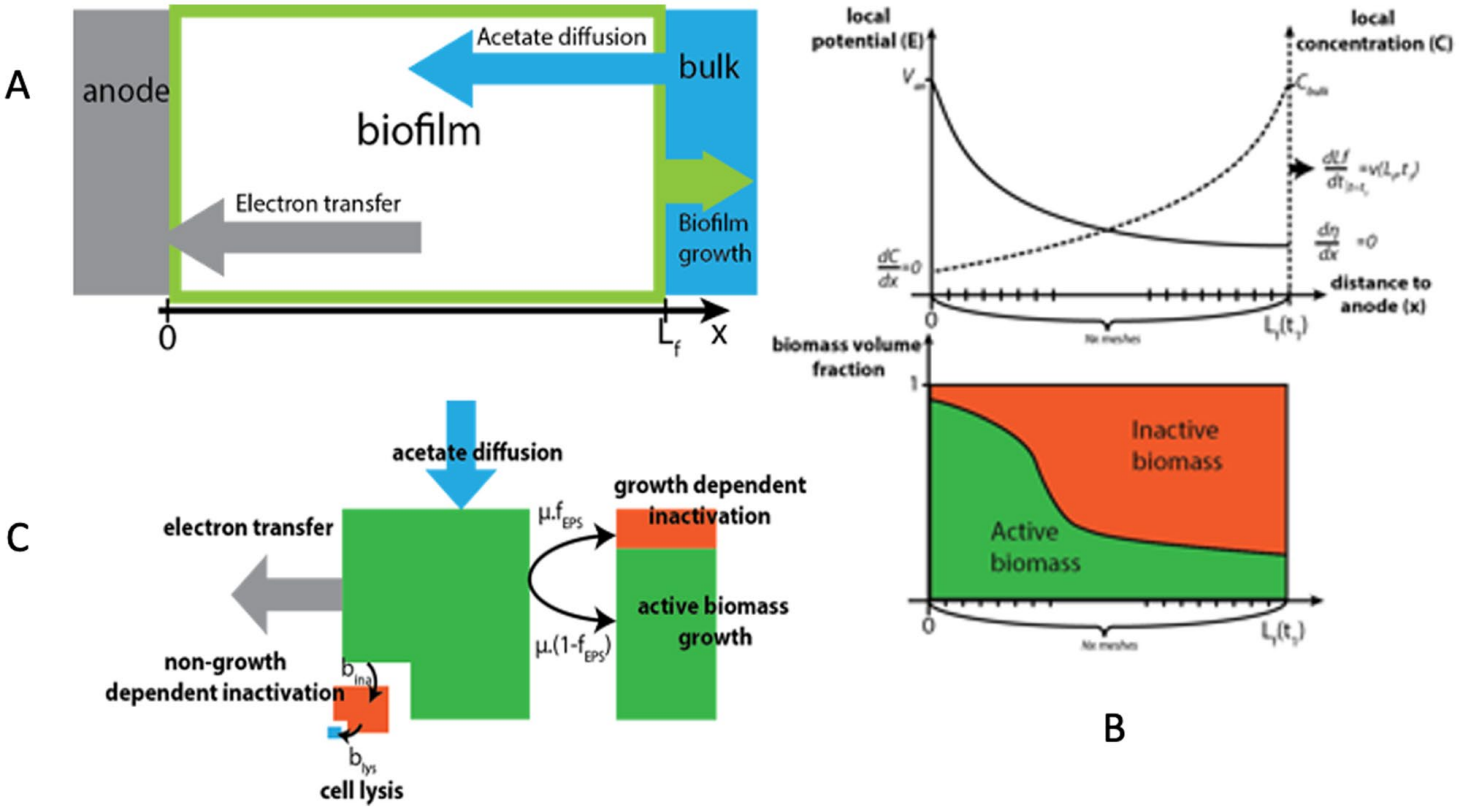
Schematic representations of model structure and concepts. (**A**) schematic 1D model representation; (**B**) schematic biomass evolution representation; (**C**) Boundaries conditions and profiles for biomass acetate concentration, local potential and volume biomass fraction.

The model aims at studying 1D growth and electron production of an anodic biofilm using a solid anode as a final electron acceptor (Fig. 1A). Two biomass volume fractions compete for space in the biofilm: an active fraction (X_a_) and an inactive fraction (X_i_).

Figure 1B, C show a schematic representation of biofilm unit volume dynamic evolution and theoretical profiles for biomass mass fraction, local potential and local acetate concentration. The X_a_ fraction represents the site of microbial metabolism where acetate is consumed and biomass is produced. The X_i_ fraction represents the extracellular polymer matrix, including pilin, OMC networks and insoluble inert biomass, resulting from two mechanisms (EPS production and cell inactivation), that both affect X_i_ properties (conductivity and density). The influence of each mechanism on biofilm activity distribution will be discussed.

### Solute concentration balance

Solute concentration is represented using a Fick law. Indeed, no convection within the biofilm is supposed and electromigration is neglected as bulk is considered as well–buffered.

1 $$\frac{{\mathrm{dC}}_{\mathrm{ac}}}{\mathrm{dt}}={\mathrm{ D}}_{\mathrm{eff},\mathrm{ac}}\frac{{\mathrm{d}}^{2}{\mathrm{C}}_{\mathrm{ac}}}{{\mathrm{dx}}^{2}}+{\mathrm{r}}_{\mathrm{ac}}$$

With the following boundary conditions:

At the anode/biofilm interface, no solutes can flow through the solid electrode:

$$\mathrm{At x}=0{:}\frac{dC}{dx}_{x=0}=0$$

At the bulk/biofilm interface, constant acetate concentration is assumed as the bulk is considered perfectly stirred reactor:

$${\text{At}}\;x = L_{f}{:}\;\;C\left( {L_{f} } \right) = C_{b,ac}$$

The source term ${\mathrm{r}}_{\mathrm{ac}}$ is related to acetate consumption rate (defined in Eq. 12).

As solutes mobility is reduced by biofilm structure, the effective diffusion coefficient (D_eff,ac_) is considered as a ratio of the bulk diffusion coefficient (commonly 0.8). However, some studies have shown that diffusion coefficient is not constant over the biofilm thickness^39^. In addition, it has been experimentally demonstrated that the effective diffusion coefficient can vary with depth in the biofilm because of the increasing density and decreasing porosity and permeability^40^.

Thus, authors proposed an empirical law to define substrate diffusion dependence on local biofilm density^41^. This equation empirically determinate for a biofilm density ranging from 0 to 400 kg m^-3^ (R^2^ = 0.819, *n* = 31) can be used to obtain a rough estimate of the effective diffusivity of a substrate for a given biofilm if the density of the biofilm is known:

2 $${\mathrm{D}}_{\mathrm{ eff},\mathrm{ ac}}={\mathrm{D}}_{\mathrm{b},\mathrm{ac}}\left(1-\frac{0.43 {{\uprho }_{\mathrm{bio}}}^{0.92}}{11.19+0.27{{\uprho }_{\mathrm{bio}}}^{0.99}}\right)={\mathrm{D}}_{\mathrm{b},\mathrm{ac}}{\mathrm{f}}_{\mathrm{Deff\,ac}}$$

where the part of the equation in brackets corresponds to the correction factor (fD_eff_._ac_) and where the local biofilm density is determined from the contribution of each volume fraction^42^, derived from the biomass balance:

3 $${\uprho }_{\mathrm{bio}}={\mathrm{X}}_{\mathrm{a}}{\uprho }_{\mathrm{a}}+{\mathrm{X}}_{\mathrm{i}}{\uprho }_{\mathrm{i}}$$

### Charge balance

As electron balance time characteristic is about 1000 times smaller as microbial growth, we assume the charge balance in steady state and use a Poisson law^43^.

4 $$0={\upsigma }_{\mathrm{bio}}\frac{{\mathrm{d}}^{2}\upeta }{{\mathrm{dx}}^{2}}+{\mathrm{r}}_{\mathrm{e}}$$

With $\upeta =\mathrm{V}-{\mathrm{E}}_{\mathrm{ka}}$ where E_ka_ represents the potential to obtain half saturated consumption rate, determined experimentally^25^. The boundary conditions used are:

- At anode/biofilm interface, the anode poised potential is set:
  $${\text{At}}\;\;x = 0{:} \;\;V_{(0)} = V_{an}$$
- At the biofilm/liquid interface, as current cannot flow out of the biofilm matrix, null ohmic potential drop is imposed:
  $$\mathrm{At }x=Lf:\,\,\, \frac{dV}{dx}_{x=Lf}=0$$

The source term r_e_ represents local current production in the biofilm and depends on conversion rate from acetate to electron (Y_e_), and thus to catabolic energy available (*cf.* Supplementary Information). Biofilm is considered as a continuous conductive structure (conduction-based biofilm model^22^), with an equivalent conductivity σ_bio_, function of the active and inactive biomass fractions. Considering a parallel circuit between X_a_ and X_i_, σ_bio_ is calculated by linear combination:

5 $${\upsigma }_{\mathrm{bio}}={\mathrm{X}}_{\mathrm{a}}{\upsigma }_{\mathrm{a}}+{\mathrm{X}}_{\mathrm{i}}{\upsigma }_{\mathrm{i}}$$

This allows to correlate the concentration of outer membranes cytochromes and pili in the EPS matrix and the EET ability.

Then, the maximum current collected at the anode/biofilm interface is the charge flux at this interface:

6 $${\mathrm{J}}_{max}={-\upsigma }_{\mathrm{bio}}\left(0\right).{\frac{\mathrm{dV}}{\mathrm{dx}}}_{x=0}$$

### Biomass balance

Biomass composition is based on local biomass volume fraction using “fuzzy layer” approach^44^.

At any times and any positions in the biofilm, the sum of all biomass is constant and validates:

7 $$\sum {X}_{i}=1$$

A convection diffusion equation is used to biomass balance in the biofilm, for both active (X_a_) and inactive (X_i_) fractions:

8 $$\frac{{\mathrm{dX}}_{\mathrm{a}}}{\mathrm{dt}}- \frac{\mathrm{d}\left(\mathrm{u}.{\mathrm{X}}_{\mathrm{a}}\right)}{\mathrm{dx}}=\left(1-{\mathrm{ f}}_{\mathrm{EPS}}\right){\mathrm{Y}}_{\mathrm{X}}{\mathrm{ r}}_{\mathrm{ac}}- {\mathrm{b}}_{\mathrm{ina}}{.\mathrm{X}}_{\mathrm{a}}={\upmu }_{\mathrm{act}}$$

9 $$\frac{{\mathrm{dX}}_{\mathrm{i}}}{\mathrm{dt}}- \frac{\mathrm{d}\left(\mathrm{u}.{\mathrm{X}}_{\mathrm{i}}\right)}{\mathrm{dx}}= \frac{{\uprho }_{\mathrm{ac}}}{{\uprho }_{\mathrm{in}}}{(\mathrm{b}}_{\mathrm{ina}}{\mathrm{X}}_{\mathrm{a}}+{\mathrm{ f}}_{\mathrm{EPS}}{\mathrm{Y}}_{\mathrm{X}}{\mathrm{ r}}_{\mathrm{ac}})- {\mathrm{b}}_{\mathrm{lys}}{\mathrm{X}}_{\mathrm{i}}={\upmu }_{\mathrm{in}}$$

X_a_ source term (µ_act_) includes two contributions: one producing active biomass from substrate (minus the investment for EPS production) an inactivation term related to the degradation of bacteria cell and apoptosis maintenance in biofilm.

X_i_ source term (µ_in_) includes three contributions: A non-growth-dependent mechanism related to conversion of active biomass into insoluble inert material, including non-biodegradable dead cell, captured suspended solid and inorganic precipitates. It is expressed as a first order to kinetic biomass conversion (X_a_).

A growth-dependent mechanism which represents EPS production investment. It is expressed as a first order to the total conversion yield (Yx), determined for active biomass production.

An inactive biomass hydrolysis rate (b_lys_), with a constant value but one order lower than endogenous decay (b_ina_). However, byproducts from cell lysis are not considered as substrate source for biofilm auto-consumption/maintenance.

Growth of the inner layers of the biofilm creates an advection movement. Therefore, we can calculate the local advection velocity *u(x)* as the sum of the contribution of each volume fraction in the inner layers.

10 $$\mathrm{u}(\mathrm{x}) ={\int }_{0}^{\mathrm{x}}{(\upmu }_{\mathrm{act}}+{\upmu }_{\mathrm{in}})\mathrm{dx}$$

Maximum biofilm thickness is defined to limit biofilm growth according to time and space differential function:

11 $$\frac{\mathrm{d}{L}_{f}}{dt}=\left(1-\frac{{\mathrm{L}}_{f}}{{\mathrm{L}}_{\mathrm{fmax}}}\right)\mathrm{u}(\mathrm{x},\mathrm{t})$$

Using this usual hindering function $\left(1-\frac{{\mathrm{L}}_{f}}{{\mathrm{L}}_{\mathrm{fmax}}}\right)$, it is no required to supply additional assumption than a maximum biofilm thickness (L_fmax_) regardless of the physical limiting factor (shear stress detachment, Red/Ox concentration gradient saturation).

### Biomass growth kinetics

**Figure 2:**
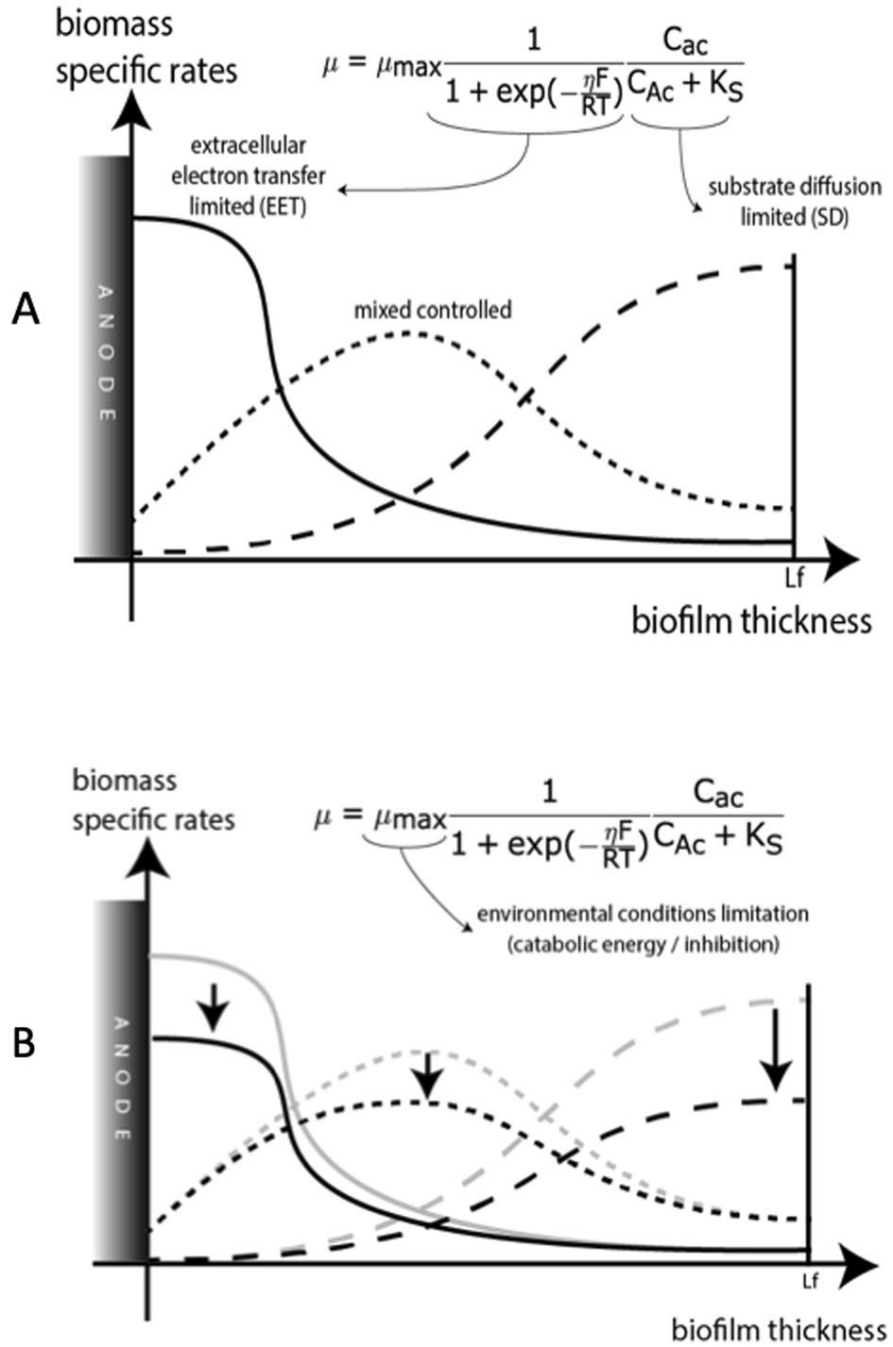
Limiting factors in the biomass specific rate inside the biofilm. (**A**) Substrate diffusion and electron transfer limitation; (**B**) Environmental conditions limitation.

A schematic representation of biomass specific profile rates can be made to illustrate the limiting processes (Fig. 2).

Acetate consumption and biomass growth specific rates in the metabolically active layer are calculated using the Nernst-Monod law^22^. This law is derived from a double Monod equation considering electron acceptor limitation directly related to local potential (Eq. 12).

12 $${\mathrm{r}}_{\mathrm{ac}}={\mathrm{X}}_{\mathrm{a}}{{\uprho }_{\mathrm{a}}\mathrm{q}}_{\mathrm{max},\mathrm{ac}}\frac{1}{1+\mathrm{exp}(- \frac{\mathrm{F}.\upeta }{\mathrm{RT}})}\frac{{\mathrm{C}}_{\mathrm{ac}}}{{\mathrm{C}}_{\mathrm{ac}}+{\mathrm{K}}_{s}}$$

It relates to metabolic pathway for extracellular electron transfer and is closely linked to biofilm development (environmental conditions, pH, anode potential, biofilm composition)^45^. In this study a value of -0.1 V (vs SHE) is considered which is in the range of results observed by non-turnover cyclic voltammetry^25,46^. Further, isothermal operation is assumed.

Three main limitation growth mechanisms can be dissociated in the Eq. (12). The first term (q_max_) variation can describe the pH inhibition phenomena. It depends on the environmental conditions and the catabolic energy available which can partially explicit the biofilm inner layer inhibition while pH drops (*cf.* thermodynamic approach in Supplementary Information) (Fig. 2B).

The second term variation represents the acceptor electron limitation (Fig. 2A). It expresses the ability of the biofilm to transfer electron to the anode and, thus depends on the midterm potential (E_ka_) and the biofilm conductivity (σ_bio_). It will be considered as extracellular electron transfer (EET) limitation. When it drives biomass growth, consumption rates will be maximized at the anode/biofilm interface, leading to a higher concentration of X_a_ in the inner layers.

The third term variation represents the donor electron limitation (Fig. 2A). It expresses the accessibility of substrate to X_a_ and, thus depends on the effective diffusion coefficient (D_eff,ac_) and the bulk concentration (C_b_ac_). It will be considered as substrate diffusion (SD) limitation. When it drives biomass growth, consumption rates will be maximum at the biofilm/liquid interface (Fig. 1B), leading to a higher concentration of X_a_ in the outer layers.

Nernst Monod equation is a relevant approach for electroactive bacteria activity in bioanode even if it cannot represent the reversible final electron transfer limitation, the “gating” (as Butler-Volmer approach do)^27^. However, it reduces the model parameters to describe electron transfer, restricted to midterm potential (E_ka_) and conductivity biofilm (*σ*_bio_). The aim of this study is first of all to show the influence of the Xi properties, and in particular the general capacity of electron transfer (intracellular and extracellular), more than to describe the transfer of electrons at the microscopic scale. In this case the approach of Nernst Monod seems sufficient.

### Thermodynamic approach for conversion yields

A thermodynamic approach^28^ is considered to evaluate acetate conversion yield into biomass and electrons. Indeed, catabolic reaction produces energy to run biomass production (anabolic reaction). However, a part of the energy is dissipated which depends mainly on substrate characteristic^47^.

Catabolic energy (∆G_cat_) corresponds to the difference between Gibbs energy of a donor couple and an acceptor couple. In this work, Acetate/HCO_3_ is the electron donor couple and the metal anode is the final electron acceptor. However, only a part of this energy is available for biomass growth since potential drop is necessary to drive electron transfer chain^45^. Intracellular redox cofactors constitute the first step of electron chain transfer to the solid anode through extracellular matrix (cytochrome network and/or pili). Hence, it drives the amount of energy that can be mobilized for biomass growth. A constant redox cofactor potential E_acc_ is considered as electron acceptor. This offers a favorable potential to run biomass growth while limiting the catabolic energy harvested. Calculations of conversion yield (Y_X_, Y_e_) and maximum specific biomass rate are detailed in Supplementary Information. All these rates integrate the part of energy for maintenance, and therefore the threshold concentration necessary to run bacteria metabolism. Then, negative growth rate of biomass could be encountered for low concentration.

### Solving method and simulation parameters

The model has been developed using finite element method software Comsol® Multiphysics. The Comsol® file is available in Supplementary Information. Simulations have been running on Intel Core Processor i5-9400H 4 Core, 8 M Cache, 2.50 GHz up to 4.3 GHz Turbo and last for less than 60 s. The biofilm thickness is represented with a 1D geometry divided in Nx meshes. Biofilm growth is simulated using a moving mesh physic with a prescribed mesh velocity at the biofilm/liquid interface (v(L_f_)). To solve the problem of numerical treatment for moving interfaces, a moving grid managed by the arbitrary Lagrangian–Eulerian method (ALE) has been used with the application of velocity limit conditions at the interfaces according to the physical quantities considered. The ALE method handles the dynamics of the deforming geometry and the moving boundaries with a moving grid with constant meshes. The algorithms of numerical resolution are represented in a diagram in the supplementary material (Fig. S1).

This study aims to describe the influence of X_i_ properties on segregation. Therefore, specific conditions, consistent with experimental conditions in steady state microbial fuel cell, are considered as summarized in Table 1. Particularly, low acetate bulk concentration (C_b,ac_) is considered in the study framework. Indeed, active/inactive segregation biofilm results of long-term experiment run in batch mode which means that local low concentrations are regularly reached in biofilm. Further, anode potential is maintained constant (V_an_ = 0.1 V Vs SHE), superior to the acetate oxidation potential (−0.276 V Vs SHE), to ensure a favorable positive potential, which is consistent with close-circuit microbial fuel cell in steady state. This allows the development of a specific biomass using a determined metabolic pathway for catabolic energy harvest (E_acc_ constant) and EET (E_ka_ constant). In supplementary information, a range of operating conditions (C_b,ac_, V_an_) have been reported in order to attest model stability and response.

**Table 1:** Summary of the parameters used in the model.

Parameters description	Symbol	Value	Units	Ref
Acetate/bicarbonate Gibbs energy	ΔG_Ac/HCO3_	−214.7	kJ.mol^-1^	Calculated
Dissipation Gibbs energy	ΔG_diss_	−439	kJ.mol^-1^	48
Acetate bulk concentration	C_b,ac_	1	mmol. L^-1^	Assumed
Bicarbonate bulk concentration	C_b,HCO3_	70	mmol. L^-1^	Assumed
Acetate diffusion coefficient	D_Ac,b_	1.1 × 10^–9^	m^-2^.s^-1^	49
Monod constant	K_s_	0.01	mmol.L^-1^	50
Midterm potential	E_ka_	−0.1	V (vs SHE)	Assumed
Intracellular acceptor electron potential	E_acc_	−0.1	V (vs SHE)	Assumed
Anode potential	V_an_	0.1	V (vs SHE)	Assumed
Inactive biomass lysis rate	b_lys_	0.01	day^-1^	Assumed

In the following analysis, Xa and Xi distributions are discussed when a steady-state is reached, after 20 days simulation (physic time).

## Results and discussion

### Fitting procedure for determination of reference conditions parameters

The comparison between the simulation results and the experimental data made it possible to define the references parameters describing the growth of the biofilm. The electroactive biofilm reference model was chosen with values initially corresponding to a thin, acetate-fed, fast-growing biofilm with Geobacter sp. as the dominant species. The growth parameters of this type of biofilm come from very reproducible results obtained at the University of Gent^50^. In order to be able to generalize the model to other types of electroactive biofilms (i.e. older, richer in EPS, etc.), a calibration was carried out by varying the conductivity; the density; EPS production and initial biomass distribution (cf. Fig. S4 in supplementary information). The chosen range of variations was determined by the values observed in the literature. The calibration was done in two steps: (1) Steady state calibration from the conductivity (*σ*) the density (*ρ*) and the production of the EPS matrix (feps); (2) Calibration in transient regime from the initial conditions of the volume fractions of biomass (inert and active). The retained values of the parameters are: *ρ* = 50 g.L^−1^; *σ* = 0.006 mS.cm^−1^ and feps = 0.01. About initial biomass distribution, the best calibration was obtained with a majority active biomass for growth (0.8–1).

### Key parameters sensitivity

Sensitivities of three key parameters (electronic conductivity, substrate’s diffusion, biofilm thickness) have been studied, in order to determine reference conditions. Reference corresponds to a mix effect limitation (substrate diffusion and electron transfer) and, therefore, we cannot observe a proper biomass segregation. Thus, under these conditions, influence of specific X_i_ properties on biomass distribution can be highlighted.

Electronic conductivity acts on biofilm viability spatial distribution. Indeed, using the Nernst Monod approach in defined conditions, it is possible to switch from substrate diffusion limitation to electron transfer limitation varying the biofilm conductivity. Potential gradient transport of charges (Ohm law) and redox electron hopping redox diffusion gradient have been compared (Supplementary Information). For high conductivity, substrate consumption rate is higher on the outer part of the biofilm which promotes biomass volume fraction segregation. Thus, X_i_ is mostly concentrated in the biofilm core (near the anode) whereas X_a_ is mostly concentrated at the biofilm liquid interface. In a second case, low conductivity creates a potential drop limiting X_a_ metabolism. Therefore, we defined a reference condition based on the intermediary mix effect to be able to represent the possible modification of biofilm activity therefore σ_bio ref_ = 0.008 mS.cm^-1^. This value is in the electrical cell property range, between outer membrane and cytoplasme, measured by electrorotation^51^. However, it’s one order smaller than biofilm conductivity measured between gold electrodes in mixed culture^52^ and pure culture^53^.

Substrate’s diffusion drives biomass growth and biofilm activity. Usually, a coefficient is used in order to reduce diffusion ability in biofilm in comparison to water. A two-order-of-magnitude change in the diffusion coefficient in the biofilm can reverse the distribution of biomass activity in the biofilm. For a low effective diffusion coefficient, substrate consumption will be limited by diffusion and, thus, mainly on the outer biofilm layer. Therefore, Xi is concentrated on the inner core whereas Xa is in the outer layer. For a high effective diffusion coefficient, it is numerically possible to switch to electron transfer limitation of the substrate consumption. However, this coefficient is one order higher than the acetate diffusion coefficient measured in water D_b ac_. It is then obvious that diffusion coefficient will not be able to invert biomass growth mode and force biofilm activity close to the anode for the operating potential V_an_.

Biofilm thickness allows to separate and evaluate the influence of electron transfer limitation and diffusion transfer limitation. For a small thickness, none of the mechanism is limiting and therefore no biomass segregation takes place into the biofilm. For a large thickness, the two mechanisms can be totally separated leading to high segregation phenomena. However, mixed culture electroactive biofilm thickness can vary from 10 to 150 µm. Thus, we defined as reference condition a 100 µm biofilm thickness to be able to observe the local segregation and to maintain a relevant size.

**Figure 3:**
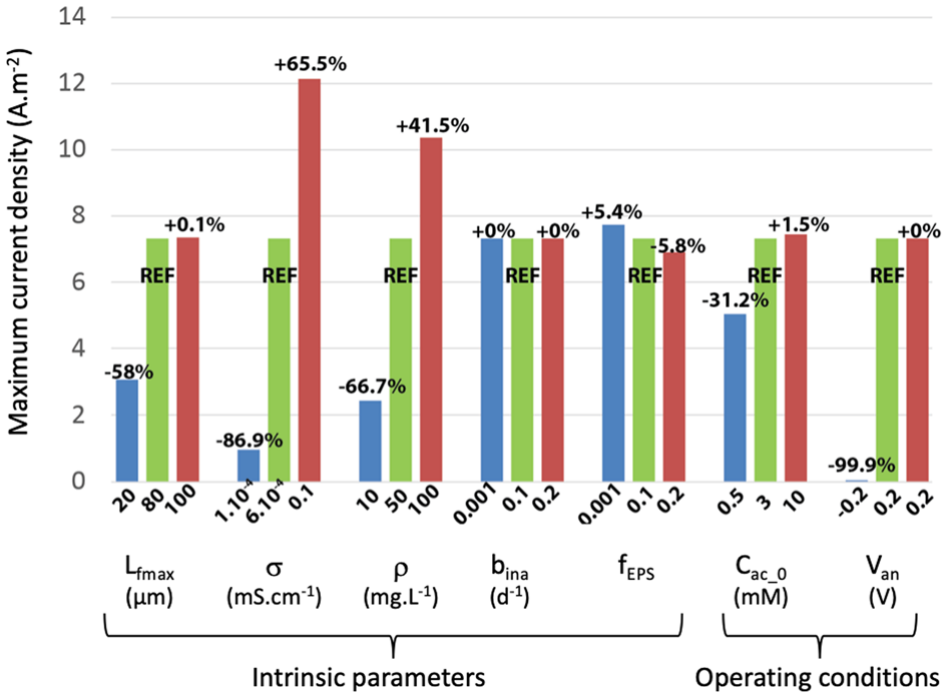
Evolution of maximum current densities with various parameters comparing to reference conditions (Table 2).

Using the model, a comparative study of density current simulated has been made with defined reference conditions (Table 2) and two extreme values chosen according literature (Fig. 3).

**Table 2:** Summary of reference conditions.

Parameters description	Symbol	Value	Units
Final biofilm thickness	L_fmax_	100	µm
Biofilm conductivity	σ_bio ref_	0.02	mS.cm^-1^
Acetate diffusion coefficient in biofilm	D_eff_,_ac_	0.77 × 10^–9^	m^-2^.Mol^-1^.s^-1^
Active biomass density	ρ_xa_	100	mg.cm^-3^
Inactive biomass density	ρ_xi_	100	mg.cm^-3^
Acetate bulk concetration	C_ac,b_	1	mmol.L^-1^
Anode potential	V_an_	0.1	V
Xi production growth dependant coefficient	f_eps_	0.1	–
Xi production growth dependant coefficient	b_ina_	0.2	d^-1^

Under reference conditions, current production is 7.4 A.m^2^. This value is slightly higher than those usually observed for experimental results in the growth of electroactive acetate biofilm under favorable conditions. This difference is due to the perfect capacity of the EET biofilm considered in the model (absence of electronic recombination, direct contact with the electrode in the whole of the biofilm and perfect homogeneity during extrapolation to the surface of the anode). But our simulation results are consistent with experimental results obtained with D. acetexigens and G. sulfurreducens under closed conditions^54^. Peak current densities of 8 to 11 A.m^-2^ (acetate concentration of 10 mM) or 7.2 to 9.9 A.m^-2^ (6 mM acetate) were observed by chronoamperometry for a longer time (200–300 h).

### Biomass distribution mechanisms

To identify the possible mechanisms of the spatial heterogeneities of the electroactive biofilms observed in some experimental studies, simulations relating to pH gradient and inactive fraction properties (density and conductivity) were carried out. The simulations were carried out with a biofilm thickness of 80 µm, an acetate concentration of 3 mM and a conductivity of 0.006 mS.cm^-1^.

### Influence of pH gradient

To take into account the influence of the pH on biofilm activity and segregation, acid–base equilibrium has been introduced (Supplementary Information). The assumption that protons accumulation in internal layers inhibits electro activity of the biofilm has been made. Two parameters are necessary, the total carbonate concentration (C_HCO3_–) and the pH value in the bulk volume (pH_bulk_).

**Figure 4:**
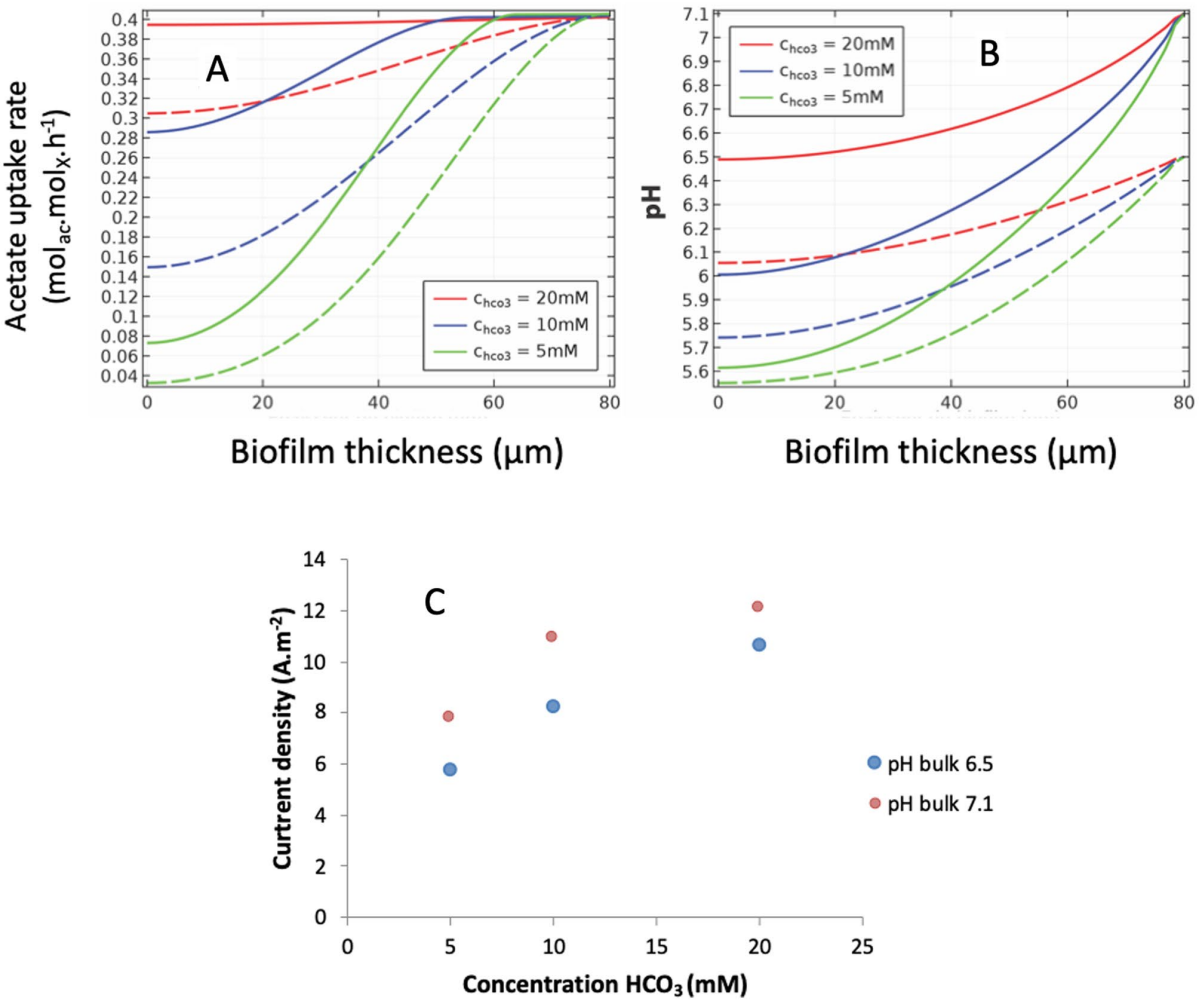
Simulation of the influence of pH on biofilm performance as a function of its thickness for acetate uptake (**A**) and pH (**B**), and total carbonate concentration for current density (**C**). Solid lines: pH bulk 6.5; Dashed lines: pH 7.1.

The influence of pH was simulated as a function of these parameters (Fig. 4).

Buffer concentrations upper than 20 mM inhibit pH gradient influence on bioanode activity. At a pH_bulk_ equal to 6.5, there is a lower current density by inhibition of metabolic activity in the inner layers (Fig. 4A, C) with a drop in local pH which tends towards a limit value (Fig. 4B) in agreement with the simulations and measurements reported by Marcus et al*.*^22^. Although pH gradient inhibits metabolism in the biofilm inner core, biomass activity segregation is not clearly observed. Relation between inactivation rate and pH should be discussed in further model description to account for biomass segregation mechanism.

### Influence of inactive fraction density (ρ_ina_)

EPS and inert product are usually considered as denser than active biomass^42^. In addition, difference of density between outside layer and the inner core of the electroactive biofilm has been reported^39^.

Two concentrations of acetate in bulk volume (C_bac_ = 0.5 mM and C_bac_ = 3 mM) are used for simulations. The lower simulates diffusion limitation and the higher a conductivity limitation with an excess of substrate.

Inactive density influence was introduced by varying the density values of the inactive fraction (*ρ*i_na_ = 20–100 g.L^-1^) and maintaining constant the density of the active fraction (*ρ*_ac_ = 50 g.L^-1^). The local density is then a function of the contribution of each of the fractions according to Eq. (3).

**Figure 5:**
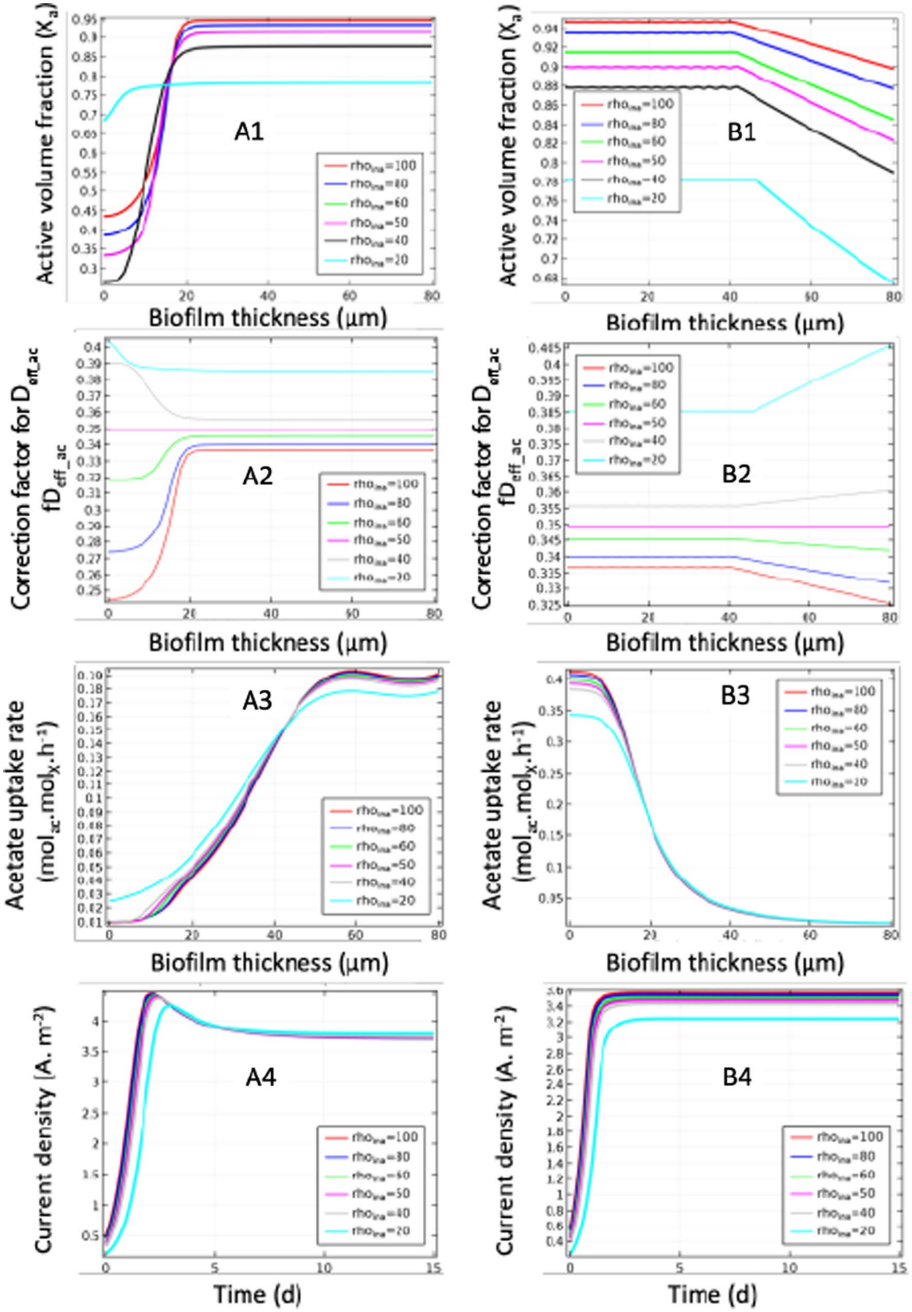
Simulations of inactive biomass density influence (rho_ina_ = *ρ*_ina_ in g.L^-1^) on biofilm performance. A1 to A4: results for limitation by diffusion; B1 to B4: results for limitation by conduction (EET) with 1: active volume fraction; 2: correction factor for D_eff_._ac_ (fD_eff_._ac_); 3: Acetate uptake rate, all vs biofilm thickness; 4: current density vs time.

The simulated parameters were the active volume fraction, the diffusion correction factor, the acetate consumption rate and the current density (Fig. 5). No influence is observed on acetate consumption rates and current densities (Fig. 5 A3–B3 and A4–B4) except for low inactive fraction density. Local segregation in the biofilm is illustrated by the active volume fraction profiles (Fig. 5 A1 and B1). Segregation is observed when growth is limited by diffusion as observed by Renslow et al.^23,39^. Low inactive fraction density (*ρ*_ina_ = 20 g.L ^-1^) leads to an increase of the diffusion correction factor (fD_eff_ac_) and thus to a homogenization of activity distribution and thus reduction of performance. In comparison, high inactive fraction density (*ρ*_ina_ > *ρ*_iac)_ reduces diffusion correction factor and the inactive fraction remains localized near the anode. This strong local heterogeneity due to a poor accessibility of the substrate in the internal layers of a thick biofilm could represent an accumulation of insoluble by-products in these layers by cell lysis^53^.

**Figure 6:**
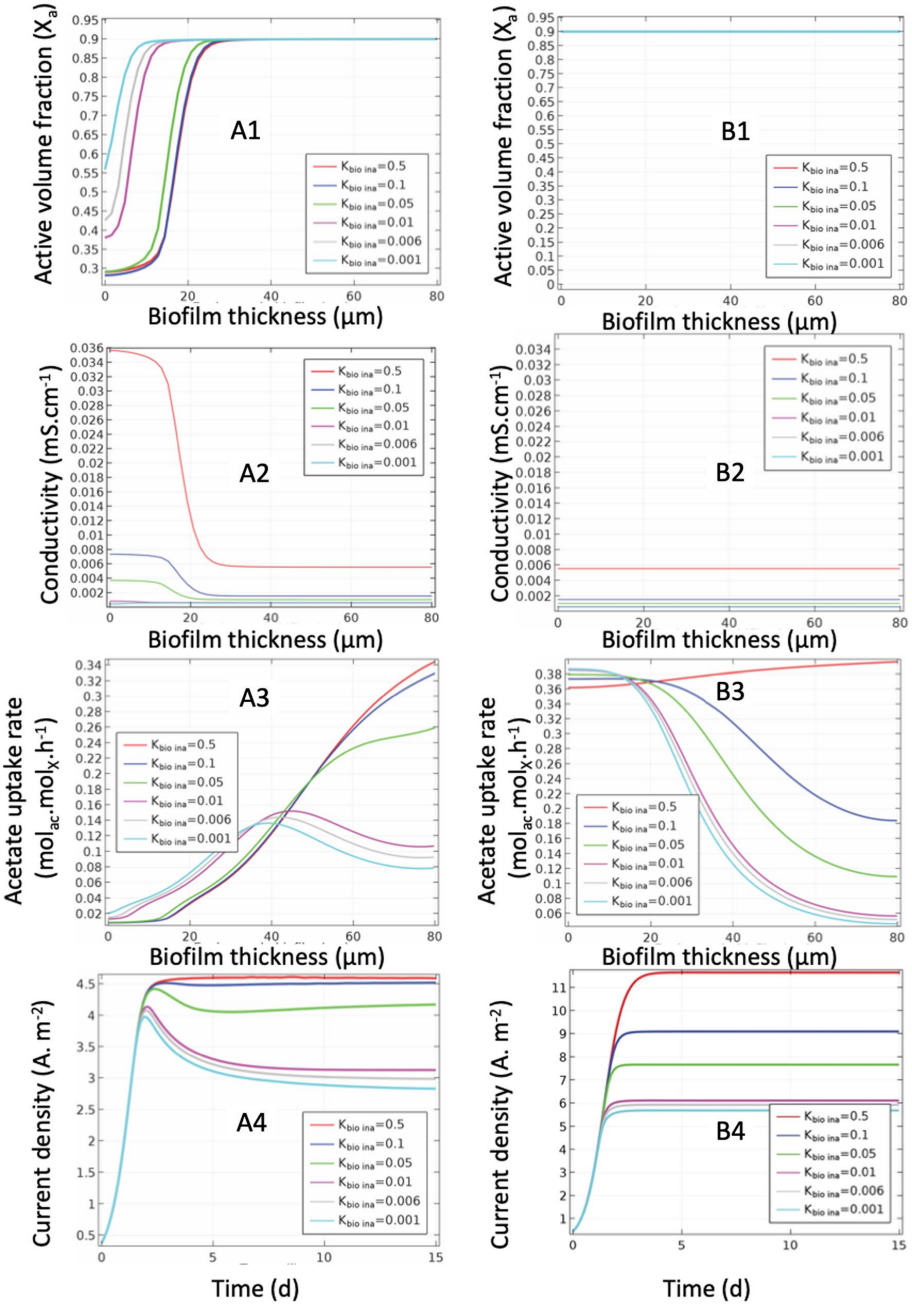
Influence inactive fraction conductivity on biofilm performance. A1 to A4: results for C_bac_ = 0.5 mM; B1 to B4: results for and C_bac_ = 3 mM with 1: active volume fraction; 2: conductivity; 3: Acetate uptake rate, all vs biofilm thickness; 4: current density vs time.

On the opposite side, when biofilm growth is limited by conductivity (Fig. 5 B1-B5), even if acetate uptake rates remain only close to the anode (0 to 20 µm), no segregation is observed on the activity distribution on the first 40 µm. In that zone, the active fraction growth and the biofilm advection are compensated by the inactivation (related to f_EPS_ and b_ina_). In the outside zone (40–80 µm), a threshold effect is observed where inactivation process becomes stronger than biofilm growth leading to a small decrease of active fraction. This changing slope (close to 40 µm) on Fig. 5 B2 and B2, is consistent with acetate uptake rate distribution (Fig. 5 B3) because the source term variation involves slope discontinuity. This behavior is due to the set of equation model (coupled equations), and as result some stable computational oscillations (FD_eff,ac_ and X_a_) could be recorded with very low magnitudes (purely numerical). In addition, the gradient of FD_eff,ac_ and X_a_ were weak and did not highlight a significant segregation: similar to homogenous distribution (Fig. 6 B1). Such small variations could not explain the inactivity of the outer layer as observed with the Live/Dead test^34^ but could justify further improvement of the model in order to couple biofilm detachment to biofilm density.

### Influence of inactive fraction conductivity (*σ*_*i*_)

The influence was determined by varying the conductivity values of the inactive fraction (*σ*_ina_ = 0.001–0.5 mS.cm^-1^) and maintaining constant the conductivity of the active fraction (*σ*_ac_ = 0.006 mS.cm^-1^). The local conductivity is then a function of the contribution of each of the fractions according to Eq. (3).

Two concentrations of acetate in bulk volume (C_bac_ = 0.5 mM and C_bac_ = 3 mM) are used for simulations. The lower simulates diffusion limitation and the higher an excess of substrate.

In both case, inactive fraction is considered as the main pathway for electron transfer and, thus, with a higher conductivity as active fraction. The role of EPS seems important because outer membrane c-type cytochrome and flavin proteins from the biofilm were involved in the electron transfer process, with the EPS acting as a transient media for the microbially-mediated EET^56^.

The simulated parameters were the active volume fraction, the biofilm conductivity, the acetate consumption rate and the current density (Fig. 6).

Segregation is only observed when the operating conditions do not allow activity throughout the thickness of the biofilm due to limitation by diffusion (Fig. 6A1). In this case, the inactive fraction is dominant near the anode and corresponds to what has been observed experimentally with studies under starvation conditions^34,57^.

The excess of substrate does not cause segregation whatever the conductivity of the inactive fraction, unlike a low concentration (Fig. 6B1). Under these conditions of concentration, the rate of consumption of the acetate (Fig. 6 B3-B4) and therefore the growth of the active fraction compensates for inactivation in the outer layer. One would expect, the same threshold effect as reported in Fig. 5B1 for thicker biofilm.

For low conductivities (≦ 0.05mS.cm^-1^), there is an inactivation of the internal layers which leads to a reduction in the current density over time until a stationary state is obtained (Fig. 6A4). But a low *σ*_*active*_ is not the only necessary condition to observe a segregation of the active fraction close to the anode as observed^33^. At the same time, a low acetate concentration is required.

Only high conductivities of the inactive fraction allow maximum activity away from the anode for thick biofilms and validate the hypothesis of an inactive conduction support fraction (EET).

In electrically conductive biofilm anodes where small potential gradient was kept, Ohmic conduction well described conductive EET with two parameters of biofilm conductivity and biofilm thickness^58^. Biofilm conductivity (responsible of EET kinetics) increase with increasing biofilm thickness implies the relationship between EPS and biofilm conductivity in electrically conductive biofilms^59^. IET mainly limits current density in the biofilm anodes, and as mentioned by Lee^58^ biofilm density of active exoelectrogens and biofilm thickness are operating parameters that can be optimized further to improve current density.

## Conclusion

We proposed a numerical model to highlight the influence of inactive fraction properties on biomass segregation in biofilm. Influence of electronic conductivity and biofilm density have been studied. Inactive fraction production rate (growth dependent or not) does not play a preponderant role in biofilm viability segregation; however, it can magnify specific properties influence. Diffusion coefficient dependence to local biofilm density is a relevant parameter to express segregation in the outer part of the biofilm. Difference between ρ_a_ and ρ_i_ enhances this phenomenon. Outer layer viability segregation can also be observed for high σ_*inactive*_. At the same time, current production increases confirming the benefit of EPS conductive investment in thick biofilm. In addition, according to the model, in specific studied conditions, the only way to obtain an X_a_ segregation close to the anode is reducing drastically mean biofilm conductivity and especially intrinsic inactive fraction conductivity two order lower than measured one. Finally, according to our simulation’s conditions, observation of high activity biofilm segregation close to the anode is not consistent with pili conductor approach. X_i_ production rate (growth dependent or non-growth dependent) is a key factor that can increase the influence of X_i_ properties. Growth dependent rate homogenizes the biomass fraction distribution and reduces current production. Non growth dependent rate forces the decay of active biomass; it’s a key model parameter to drive biomass segregation. However, role of inert biomass in electroactive biofilm still needs to be clarified and inactivation rates should depend on external factors (pH, substrate depletion or electron transfer intensity). More active biomass characterization using a well detailed and reproducible protocol using poised potential and continuous flow convection must be performed in order to validate this numerical approach.

## Supplementary Information

Supplementary Information.

## List of symbols

b_ina_: Inactivation coefficient [/day]
b_lys_: Cell lysis rate [/day]
C_ac_: Biofilm acetate concentration [mol/L]
C_b,ac_: Acetate concentration in bulk [mol/L]
C_b,HCO3_: Bicarbonate concentration in bulk [mol/L]
D_b,ac_: Bulk acetate diffusion coefficient [m^2^/s]
D_eff,ac_: Biofilm efficient acetate diffusion coefficient [m^2^/s]
ΔG_ana_: Anabolic Gibbs Energy [J/mol X_a_]
ΔG_cat_: Catabolic Gibbs Energy [J/mol X_a_]
ΔG_diss_: Dissociation Gibbs Energy [J/mol X_a_]
E_ka_: Mid term potential [V]
η: Local overpotential [V] (= V−E_ka_)
F: Faraday constant [s.A/mol]
f_cat_: Number of catabolic reaction cycle to run metabolism [−]
f_eps_: EPS production coefficient [−]
${\mathrm{J}}_{max}$: Current density at anode/biofilm interface [Am^-2^]
k: Density coefficient variation [mg/cm^3^]
K_s_: Monod constant [mol/L]
L_fmax_: Maximum biofilm thickness [µm]
m_G_: Maintenance energy [J/mol X]
m_ac_: Maintenance energy [J/mol Ac]
μ_max_: Maximum biomass specific rate [/s]
Μ: Biomass specific rate [/s]
q_max, gibbs_: Maximum Energy consumption rate [kJ/mol X_a_ /s]
q_max, ac_: Maximum acetate consumption rate [mol Ac/mol X_a_ /s]
r_ac_: Acetate consumption rate [mol e^-^/mol X_a_ /s]
r_e_: Electron production rate [mol Ac/mol X_a_ /s]
R: Gas constant [J/mol/K]
ρ_bio_: Local biofilm density [mg/cm^3^]
ρ_a_: Active biomass density [mg/cm^3^]
ρ_i_: Inactive biomass density [mg/cm^3^]
σ_bio_: Local biofilm conductivity [mS/cm]
σ_a_: Active biomass conductivity [mS/cm^3^]
σ_i_: Inactive biomass conductivity [mS/cm^3^]
t: Time [s]
T: Temperature [K]
u: Local advection velocity [m/s]
V: Anode potential [V]
V_an_: Operating anode potential [V]
X_a_: Biomass active fraction [−]
x: Space abscise [m]
X_i_: Biomass inactive fraction [−]
Y_X_: Conversion yield into biomass [mol X_a_/mol Ac]
Y_e_: Conversion yield into electron [mol e^-^/mol Ac
