# Supplementary material for: Characterization of spatiotemporal electroactive anodic biofilm activity distribution using 1D simulations
Source: Sci Rep. 2022 Apr 7;12:5849. doi: 10.1038/s41598-022-09596-w (PMC8990003; doi:10.1038/s41598-022-09596-w)
Supplement: Supplementary file 1 — Supplementary Information. [file 41598_2022_9596_MOESM1_ESM.docx]

**Supplementary material: Characterization of spatiotemporal electroactive anodic biofilm activity distribution using 1D simulations.**

**Pierre Belleville^1,2^, Gerard Merlin^1,2^, Julien Ramousse^2^, and Jonathan Deseure^1*^**

**^1^**Univ. Grenoble Alpes, Univ. Savoie Mont Blanc, CNRS, Grenoble INP, LEPMI, 38000 Grenoble, France

**^2^**Univ. Savoie Mont-Blanc, CNRS, LOCIE, UMR 5271, Polytech Annecy, Chambéry, bât. Helios, 60 rue du lac Léman, Savoie Technolac, 73370 Le Bourget du Lac

**Thermodynamic calculation of conversion yields and maximum specific biomass growth**

Gibbs energy of electron donor couple is calculated from the following equation:

${C_{2}H_{3}O_{2}}^{-}+ 4H_{2}O \to2{\mathrm{HC}O_{3}}^{-}+9H^{+}+{8e}^{-} (14)$ (Y_cat_)

Catabolic Gibbs energy is corrected with acetate, bicarbonate and protons concentration in the biofilm. However, as the reactor is well buffered, there is no pH gradient and bicarbonate and protons concentrations remain constant.

$${\Delta G}_{\mathrm{don}}={\Delta G}_{{C2H3O2}/{HCO3}}^{0}+RT\ln\left( {(\frac{C_{\mathrm{ac}}}{C_{\mathrm{ac}}^{\theta^{'}}})}^{-1}.{(\frac{C_{b,HCO3}}{C_{HCO3}^{\theta^{'}'}})}^{2}({\frac{C_{b,H}}{C_{H}^{\theta^{'}}})}^{9} \right)$$

Gibbs energy of electron acceptor is based on mid-term potential E_ka_.

$${\Delta G}_{\mathrm{acc}}=-8FE_{\mathrm{acc}}$$

$${{\Delta G}_{\mathrm{cat}}=\Delta G}_{\mathrm{don}}-{\Delta G}_{\mathrm{acc}}$$

Anabolic reaction is equilibrated using the accepted biomass composition (CH_1.8_O_0.5_N_0.2_)

$0.525{C_{2}H_{3}O_{2}}^{-}+0.2NH_{4}+0.275H^{+}\to CH_{1.8}O_{0.5}N_{0.2}+0.4H_{2}O$ (Y_an_)

Catabolic energy is used to run anabolic energy. In this transformation a part of the energy is dissipated. Hence, we can determine the catabolic factor:

$$f_{\mathrm{cat}}=\frac{{\Delta G}_{\mathrm{diss}}^{\max}+{\Delta G}_{\mathrm{an}}}{{\Delta G}_{\mathrm{cat}}}$$

The overall growth reaction (OGR) conversion yields for one mole of biomass can be then derived from the main expression

$$Y_{OGR}=Y_{an}+f_{cat}Y_{cat}$$

$$-(f_{\mathrm{cat}}+0.525){C_{2}H_{3}O_{2}}^{-}-0.2NH_{4}-\left( 4f_{\mathrm{cat}}-0.4 \right)H_{2}O$$

$+CH_{1.8}O_{0.5}N_{0.2}+{2f_{\mathrm{cat}}HC0}_{3}+\left( 9f_{\mathrm{cat}}-0.275 \right)H^{+}+{8f}_{\mathrm{cat}}e^{-}=0$

We can explicit the stoichiometric coefficient per mole of acetate:

$$Y_{x}=\frac{1}{f_{\mathrm{cat}}+0.525}; Y_{e}=\frac{8f_{\mathrm{cat}}}{f_{\mathrm{cat}}+0.525}$$

Considering the hypothesis of the limiting electron transfer rate in microbial electron chain [64]and maintenance energy correlation, an estimation of maximum energy consumption rate and maintenance term is done.

$$q_{\max}^{\mathrm{Gibbs}}=-3\frac{{\Delta G}_{\mathrm{cat}}}{8}exp(-\frac{69000}{R}\left( \frac{1}{T}-\frac{1}{298} \right))$$

$$m_{ac}=\frac{m_{G}}{{\Delta G}_{\mathrm{cat}}}=-4.5 exp(-\frac{69000}{R}\left( \frac{1}{T}-\frac{1}{298} \right))$$

It’s then possible to extend maintenance needs for other compounds such as electron.

$$m_{e}= Y_{cat,e}m_{\mathrm{ac}}=8 m_{\mathrm{ac}}$$

Applying the Herbert-Pirt relation, we can define the maximum biomass specific rate and then the maximum acetate consumption rate:

$$\mu_{max}=\frac{q_{\max}^{\mathrm{Gibbs}}-m_{G}}{{\Delta G}_{\mathrm{diss}}^{\max}}=-\frac{3\frac{{\Delta G}_{\mathrm{cat}}}{8}+4.5}{{\Delta G}_{\mathrm{cat}}}\exp\left( -\frac{69000}{R}\left( \frac{1}{T}-\frac{1}{298} \right) \right)$$

$$q_{max,ac}=-\left( f_{\mathrm{cat}}+0.525 \right)\mu_{\max}+m_{\mathrm{ac}}$$

$$\mu=Y_{X}\left( r_{\mathrm{ac}}-m_{ac} \right)$$

It’s finally possible to determine the local rate of production of electrons:

$$r_{e}=X_{a}.\rho_{a}.F.{(Y}_{e}.\mu-m_{e})$$

**Conceptual model description**

Supplementary Figure S1: Conceptual diagram of the algorithms used in **COMSOL Multiphysics.**

**Equation’s systems**

1. **Equation’s system for Reference model (conduction)**

| Variable | Equations | Boundary conditions | | Initial conditions |
| --- | --- | --- | --- | --- |
|  |  | Y=0 | Y=Lf |  |
| Source term  r_ac_ | $\mu=\mu_{max}\frac{C_{ac}}{C_{ac}+K_{ac}}\frac{1}{\begin{aligned} 1+\exp\left( -\frac{F\left( V-E_{ka} \right)}{RT} \right) \\ r_{ac}=Y_{X}c_{X}X_{a}\mu\\ r_{e^{-}}=FY_{e}-Y_{X}c_{X}X_{a}\mu=FY_{e}-Y_{X}\frac{\rho_{X}}{M}X_{a}\mu\end{aligned}}$ |  |  |  |
| Soluble species  C_i_ | $\frac{dC_{ac}}{dt}=D_{eff,ac}\frac{dC_{ac}}{dt}+r_{ac}$ | $\frac{dC_{ac}}{dy}=0$ | $C_{ac}=C_{ac0}$ | $C_{ac}=C_{ac0}$ |
| Potential V | $0=\sigma_{bio}\frac{d^{2}(V-E_{ka})}{dy^{2}}+r_{e^{-}}$ | $V=V_{an}$ | $\frac{dV}{dy}=0$ | $V=V_{an}$ |
| Current density | $j=\sigma_{bio}\frac{d(V-E_{ka})}{dy}$ |  |  |  |
| Volume fraction | $X_{a}+X_{in}=1$ |  |  |  |
| Active biomass | $\frac{dX_{a}}{dt}-D_{X}\frac{d^{2}X_{a}}{dy^{2}}-\frac{d\left( v{.X}_{a} \right)}{dx}=r_{a}$  $=\left\{ \begin{aligned} \left( 1-f_{EPS} \right)c_{X}X_{a}\mu_{Xa} ifC_{i}>C_{minXin} \\ -b_{ina}c_{X}X_{a} ifC_{i}<C_{minXin} \end{aligned} \right\}$ | $\frac{dX_{a}}{dy}=0$ | $\frac{dX_{a}}{dy}=0$ | $X_{a}=0.5$ |
| Inactive biomass | $\frac{dX_{in}}{dt}-D_{X}\frac{d^{2}X_{in}}{dy^{2}}-\frac{d\left( v{.X}_{a} \right)}{dx}=r_{Xin}$  $=\left\{ \begin{aligned} f_{EPS}c_{X}X_{a}\mu_{Xa} ifC_{i}>C_{minXa} \\ b_{ina}c_{X}X_{a} ifC_{i}<C_{minXa} \end{aligned} \right\}$ | $\frac{dX_{in}}{dy}=0$ | $\frac{dX_{in}}{dy}=0$ | $X_{in}=0.5$ |

1. **Equation’s system for RedOx model**

| Variable | Equations | Boundary conditions | | Initial conditions |
| --- | --- | --- | --- | --- |
|  |  | Y=0 | Y=Lf |  |
| Source term  r_i_  (substituted) | $\mu=\mu_{max,Xi}\frac{C_{i}}{C_{i}+K_{S,Xi}}\frac{C_{ox}}{\begin{aligned} C_{ox}+K_{ox} \\ r_{i}=Y_{i}c_{X}X_{a}\mu\\ =Y_{i}\frac{\rho_{X}}{M}X_{a}\mu\end{aligned}}$ |  |  |  |
| Redox balance  (added) | $C_{red}+C_{ox}=C_{T}$ |  |  | $C_{red}=C_{ox}=\frac{C_{T}}{2}$ |
| Maximum growth  (instead, current density) | $\frac{dL_{f}}{dt}=v(L_{f})-v(L_{f})\frac{C_{red}}{C_{T}}$  $j_{X}=C_{T}D_{e}^{1/2}\delta\frac{dC_{red})}{dy}$ |  | $\frac{dC_{red}}{dy}=0$ |  |
| Final transfer  (added) | $J=k_{0}X_{a}\exp\left[ C_{ox}\exp\left( -\alpha\frac{F\left( V_{an}-E_{redox} \right)}{RT} \right)-C_{red}\exp\left( \left( 1-\alpha\right)\frac{F\left( V_{an}-E_{redox} \right)}{RT} \right) \right]$ | | |  |

1. **Equation’s system to take into account influence of pH**

| Variable | Equations | Boundary conditions | | Initial conditions |
| --- | --- | --- | --- | --- |
|  |  | Y=0 | Y=Lf |  |
| Soluble species  C_i_ (substituted) | $\frac{dC_{i}}{dt}=D_{eff,i}\frac{d^{2}C_{i}}{dy^{2}}+\frac{z_{i}D_{eff,i}F}{RT}\frac{dC_{i}}{dy}+r_{i}+r_{i ab}$ | $\frac{dC_{i}}{dy}=0$ | $C_{i}=C_{b}$ | $C_{i}=C_{b}$ |
| Neutrality  (added) | $\sum z_{i}C_{i}=C_{r}$ |  |  | $C_{red}=C_{ox}=\frac{C_{T}}{2}$ |
| Acid-base reactions  (added) | $r_{OH^{-}ab}=-r_{H^{+}}=k_{H_{2}Oab}\left( 1-\frac{C_{H^{+}}C_{{OH}^{-}}}{K_{H_{2}Oab}} \right)$  $r_{HCO_{3}^{-}ab}=-r_{CO_{2}}=k_{{CO}_{2}Oab}\left( 1-\frac{C_{H^{+}}C_{HCO_{3}^{-}}}{K_{{CO}_{2}Oab}} \right)$  $r_{Ac^{-}ab}=-r_{AcH}=k_{Ac^{-}ab}\left( 1-\frac{C_{H^{+}}C_{{Ac}^{-}}}{K_{AcH ab}} \right)$ |  |  |  |
| Source term  r_i_  (substituted) | $q_{max ab}=0 pH<5.5$  $q_{max ab}=\sin\left( \left( pH-5.5 \right).\frac{\pi}{2} \right)q_{max ac} 5.5<pH>6.5$  $q_{max ab}=q_{max ac} pH>6.5$ |  |  |  |

**Comparison Redox / macroscopic election conduction:**

In the reference model, the extracellular transfer mode of electrons is a metallic conduction type.

We have modified this model to simulate a transfer by conduction of a RedOx type. We assume for this that the final transfer of electrons is not limiting and perfectly reversible. We thus reduce the model proposed by Strycharz-Glaven et al^1^ to three parameters: the total concentration of charge carriers (C_T_); the apparent coefficient diffusion of charges (D_e_) and the maximum thickness of the biofilm (L_fmax_).

The current densities were simulated by the RedOx model for a set of values of C_T_ and D_e_ (Fig. S2). It is observed that the Randles-Sevcik law is respected with a scan speed characteristic of the concentrations. The RedOx model therefore makes it possible to simulate the current produced when the outer layer is saturated with reduced cofactors.

Supplementary Figure S2: Study of the simulated current densities as a function of the diffusion coefficient and the concentrations of charge carriers.

With reference model, all the biofilm was active for a thickness less than 60µm (Fig. S3A) but with RedOx model, at low conductivity we observe a heterogeneity due to inactivation of external layer. It is then noted that for low conduction values with the reference model, the current density is limited as a function of the conductivity and the thickness of the biofilm (Fig. S3B). In order to obtain a biomass fully active for a thickness of 100 µm, a high conductivity is then required.

Supplementary Figure S3: Comparison of the RedOx model with the reference model according to the thickness of the biofilm. A: Fraction of active biomass; B: Current densities.

Even if the RedOx model allows a coherent simulation of an electroactive biofilm with the experimental measurements, equivalent simulations can be obtained with the reference model which is easier to implement.

**Model calibration**

Supplementary Figure S4: Calibration for the simulation of the current density according to different experimental parameters. A: Influence of conductivity at ρ= 50 g.L^-1^ and f_eps_= 0.1; B: Influence of density at σ=0.01 mS.cm^-1^; C: Influence of EPS production coefficient at ρ= 50 g.L^-1^ and σ=0.01 mS.cm^-1^; D: Influence of initial biomass distribution at ρ= 50 g.L^-1^, σ=0.006 mS.cm^-1^ and f_eps_= 0.01; E: Determination of σ, ρ and f_eps_ to obtain maximum current density; F: Final calibration with simulate data (solid line) and experimental data (dotted line).

**Influence of V_an_ on biofilm activity under reference conditions**


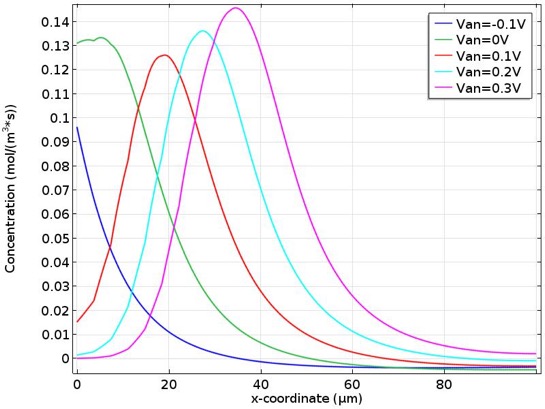


Figure S5: Active fraction production rates in biofilm function of **V_an_**


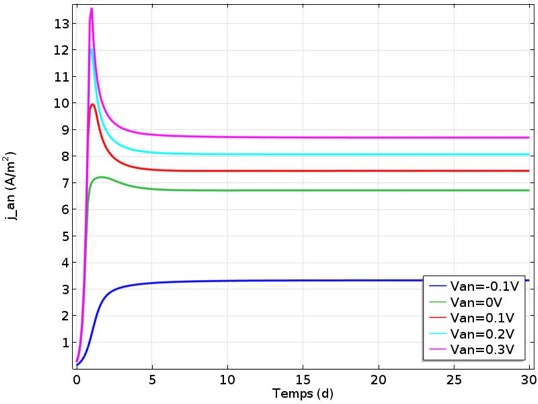


Figure S6: Current production in time function of **V_an_**


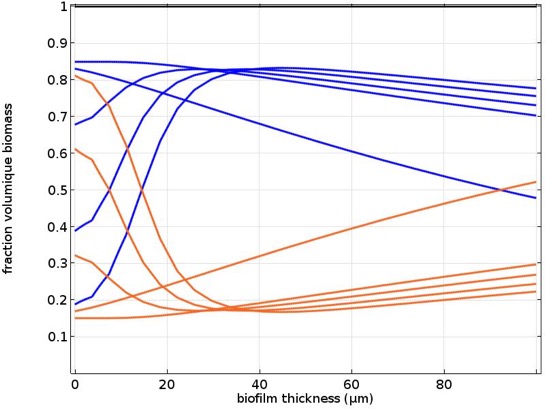


V_an_ up

V_an_ up

Figure S7: biomass fraction (red: inactive, blue: active) biofilm function of **V_an_**

**Influence of C_b ac_ on biofilm activity under reference conditions**

**
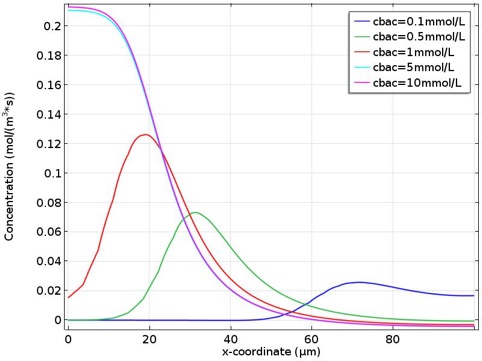
**

Figure S8: Active fraction production rates in biofilm function of **C_b ac_**

**
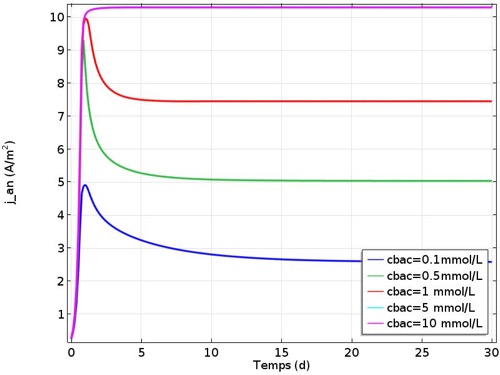
**

Figure S9: Current production in time function **C_b ac_**

**
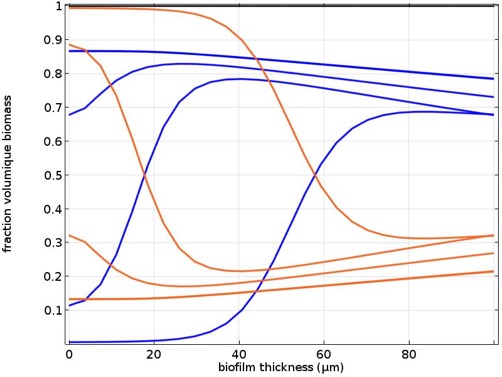
**

c_b ac_ up

c_b ac_ up

Figure S10: biomass fraction (red: inactive, blue: active) biofilm function of **C_b ac_**

# ****Supplementary Bibliography****

1. Strycharz-Glaven, S. M. & Tender, L. M. Reply to the ‘Comment on “On electrical conductivity of microbial nanowires and biofilms”’ by N. S. Malvankar, M. T. Tuominen and D. R. Lovley. Energy Environ. Sci., 2012, 5*.* *Energy Environ. Sci.* **5**, 6250-6255 (2012).
